# Comparative Evaluation of the Staining Resistance of Two Single-Shade Composites in Coffee and Chlorhexidine: A Spectrophotometric Analysis

**DOI:** 10.7759/cureus.82548

**Published:** 2025-04-18

**Authors:** Unmesh Khanvilkar, Shrinath D Kulkarni, Siddhesh Bandekar, Ved M Talathi, Oshin Baghel, Priyanka Razdan, Seema Gupta

**Affiliations:** 1 Department of Microdentistry, Maharashtra University of Health Sciences Regional Centre, Mumbai, IND; 2 Department of Conservative Dentistry and Endodontics, Yogita Dental College and Hospital, Khed, IND; 3 Department of Conservative Dentistry and Endodontics, Rishiraj College of Dental Sciences and Research Center, Bhopal, IND; 4 Department of Paediatric and Preventive Dentistry, Yogita Dental College and Hospital, Khed, IND; 5 Department of Orthodontics, Kothiwal Dental College and Research Centre, Moradabad, IND

**Keywords:** chlorhexidine, coffee, composite resins, resistance, spectrophotometry, staining

## Abstract

Introduction: Color stability is a critical factor for the longevity of composite restorations, especially in esthetic dentistry. Single-shade universal composites have been designed to blend seamlessly with natural tooth structures. This study aimed to compare the staining resistance of Omnichroma (Tokuyama Dental, Tokyo, Japan) and Vittra Unique (FGM Dental Group, Joinville, Santa Catarina, Brazil) over a 30-day period using spectrophotometric analysis to assess color changes. The null hypothesis was that no significant difference would be observed between the two composites in terms of staining resistance.

Materials and methods: An in vitro study was conducted using 40 disc-shaped composite specimens divided into two groups: Group A (Omnichroma) and Group B (Vittra Unique). Each group was further divided into two subgroups based on the staining solution used (coffee and 0.2% chlorhexidine). The specimens were prepared using standardized molds, light-cured, polished, and stored in artificial saliva for 24 hours prior to immersion. Each specimen was exposed to its respective staining solution for eight hours daily for 30 days, followed by rinsing and solution replacement. Thermal cycling was performed to simulate the oral conditions. Quantification of color alteration (ΔE values) was done using a spectrophotometer (Vita Easyshade 2.0, VITA Zahnfabrik, Germany) at baseline and after 30 days. Statistical analysis was conducted using independent t-tests and analysis of variance (ANOVA), with a significance level of p < 0.05.

Results: A significant difference in staining resistance was observed between groups (p = 0.001). Vittra Unique exhibited higher ΔE values than Omnichroma, indicating greater discoloration. The highest color change was found in the Vittra coffee subgroup (ΔE = 2.70 ± 0.56), while the lowest was in the Omnichroma coffee subgroup (ΔE = 1.25 ± 0.17). Chlorhexidine also caused staining, albeit to a lesser extent than coffee did.

Conclusion: Omnichroma demonstrated superior resistance to staining compared with Vittra Unique, particularly against coffee-induced discoloration. These findings suggest that Omnichroma may be a more suitable choice for patients frequently exposed to staining agents. Further research is recommended to explore the long-term color stability under varying conditions.

## Introduction

The esthetic appearance of teeth plays a pivotal role in modern dentistry, significantly influencing patient satisfaction and psychological well-being. A bright, natural smile is often associated with health, vitality, and attractiveness, making the preservation of tooth color a critical aspect of restorative dentistry [1]. However, tooth discoloration remains a prevalent challenge due to the esthetic outcome of dental restorations and necessitating frequent replacements or repairs [2]. Discoloration can stem from a variety of etiological factors and is broadly categorized as extrinsic, intrinsic, or a combination of both. Extrinsic factors include the consumption of chromogenic substances, such as coffee, tea, red wine, and certain medications, while intrinsic factors may involve aging, trauma, or systemic conditions affecting enamel and dentin [3].

Among restorative materials, composite resins are widely utilized because of their excellent esthetic properties and versatility; however, their susceptibility to staining over time compromises their long-term performance and esthetic integrity. Composite restorations are particularly prone to staining because of their organic matrix composition [4]. The primary factors contributing to composite staining include surface roughness, water sorption, and chemical interactions between the resin matrix and staining agents [4]. Surface irregularities can harbor pigments, whereas water sorption facilitates the penetration of hydrophilic chromogens into the material. Furthermore, the degree of monomer conversion and the presence of unreacted double bonds can exacerbate susceptibility to discoloration, as these chemical vulnerabilities allow for interactions with external agents [5].

Commonly used substances such as coffee and antimicrobial mouthwash such as chlorhexidine, which are frequently used in oral hygiene protocols, are notable culprits in composite staining. Coffee contains polyphenolic compounds such as tannins, which possess strong chromogenic potential because of their ability to adsorb onto the resin surface and penetrate the polymer network [6]. Chlorhexidine, a widely used antimicrobial agent, induces staining owing to its cationic nature, which promotes binding to anionic sites on the composite surface, forming stable pigmented complexes over time. These chemical interactions underscore the need for materials with enhanced resistance to discoloration [7].

Various methods have been employed to assess the color stability of dental composites, including visual assessment, colorimetry, and spectrophotometry [8]. Visual assessment, while simple, is highly subjective and influenced by observer bias, lighting conditions, and individual perception, rendering it unreliable for precise evaluations. Colorimetry provides a more objective measure by quantifying color; however, it is limited by its inability to account for spectral reflectance across the full visible spectrum [9]. In contrast, spectrophotometry offers superior reliability by measuring the reflectance or absorbance of light at multiple wavelengths, providing a comprehensive and reproducible analysis of color changes. Its precision, consistency, and ability to detect subtle shifts in hue, chroma, and lightness make it the gold standard for evaluating staining resistance in dental materials, ensuring accurate comparisons under standardized conditions [8].

Despite advancements in composite technology, including the development of single-shade composites such as Omnichroma (Tokuyama Dental, Tokyo, Japan) and Vittra Unique (FGM Dental Group, Joinville, SC, Brazil), which aim to simplify shade matching and improve esthetic outcomes, the literature reveals significant research gaps [10,11]. Most studies have focused on traditional multi-shade composites, with limited comparative data on the staining resistance of single-shade systems under prolonged exposure to clinically relevant staining agents, such as caffeine and chlorhexidine [12]. Additionally, the chemical and physical mechanisms underlying staining in these newer materials remain underexplored, particularly under simulated real-world conditions over extended periods. The lack of standardized protocols for evaluating staining resistance further complicates the interpretation of existing findings, leaving clinicians with insufficient evidence to select proper material for patients with high esthetic demands when exposed to staining agents. This study sought to fill these voids by performing a comparative analysis of the staining resistance exhibited by Omnichroma and Vittra when exposed to coffee and chlorhexidine over a duration of 30 days, employing spectrophotometry to guarantee accurate and dependable assessments of color stability. The null hypothesis formulated for this research asserted that there would be no statistically significant difference in staining resistance between the examined groups.

## Materials and methods

Study design and setting

This in vitro study was conducted in the Department of Conservative Dentistry and Endodontics, Yogita Dental College, Khed, Maharashtra, India, from January to February, 2025, to evaluate the staining resistance of two single-shade universal composites. The study was conducted in a controlled laboratory setting to ensure consistency in sample preparation, staining procedures, and spectrophotometric analysis. Owing to the utilization of disc-shaped composite specimens in the research, the necessity for informed consent and approval from the ethics committee was deemed inapplicable.

Sample size calculation

The sample size was determined using G*Power statistical software version 3.6.9 (Heinrich-Heine-Universität Düsseldorf, Düsseldorf, Germany), with a confidence level of 95%, statistical power of 80%, and an effect size (f) of 0.89, which was derived from a previous study [13]. The calculations indicated that 32 specimens were required to compare two independent means, with eight specimens per subgroup. To minimize the risk of specimen loss during the experiment, the study design was adjusted to include 10 specimens in each subgroup, resulting in a total of 40 specimens divided equally between the two composite materials.

The inclusion criteria for the study were single-shade universal composite materials with reported chameleon effects, which were selected based on their widespread use in esthetic dentistry. The exclusion criteria were any composite materials that required additional layering for shade matching or those lacking documented color stability data. Only fresh and unexpired composite materials were used to maintain the reliability of the results.

Methodology

**Figure 1:**
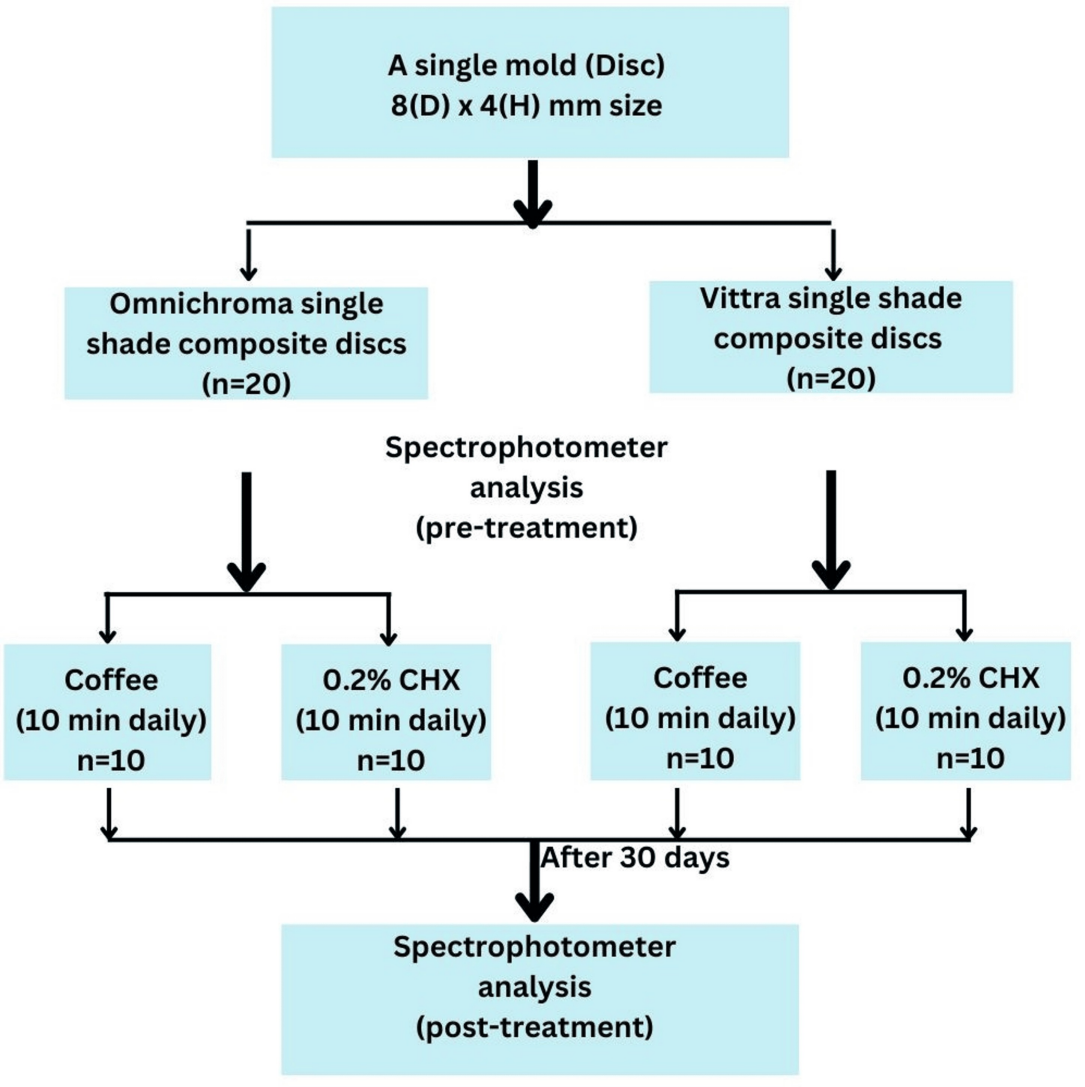
Study design. D: diameter, H: height, CHX: chlorhexidine, Omnichroma (Tokuyama Dental, Tokyo, Japan), Vittra Unique (FGM Dental Group, Joinville, SC, Brazil).

Forty composite resin discs were fabricated, and 20 samples were prepared for each group. Group A (Omnichroma) and Group B (Vittra Unique). Each disc was standardized to 8 mm in diameter and 4 mm in height using the cylindrical molds made of Teflon (polytetrafluoroethylene). The composites were packed into molds in 2-mm increments using handheld instruments. Excess composite material was eliminated by attaching a strip of transparent adhesive tape to the resin composites and exerting manual pressure through a 1-mm-thick glass coverslip. Light curing was performed using a light-emitting diode (LED) curing unit (Bluephase G2, Ivoclar Vivadent, Liechtenstein) with an intensity of 1200 mW/cm² for 20 seconds by lightly touching the tip of the LED unit on the glass coverslip. The specimens were subsequently preserved in artificial saliva solution (Hypozalix, Biocodex, France) for 24 hours at a controlled temperature of 37^ o^C in an incubator (Dentsply Sirona Inc., Charlotte, North Carolina, United States) to facilitate comprehensive polymerization. After curing, the specimens were finished by a single experienced and calibrated endodontist following the manufacturer’s instructions. The superior surfaces of each specimen were meticulously polished using polishing disks (Sof-Lex™, 3M Company, Saint Paul, Minnesota, United States) in conjunction with a high-speed handpiece to remove surface irregularities and ensure uniform smoothness (Figure 1).

After fabrication, each group was further subdivided into two subgroups based on the staining solution used: coffee solution and 0.2% chlorhexidine (Hexidine; ICPA Health Products Ltd., Mumbai, India). Each subgroup consisted of 10 specimens. The staining solutions were freshly prepared and stored in sealed containers. The coffee solution was formulated by mixing 10 g of coffee (Nescafé Original; Nestlé S.A., Vevey, Switzerland) with 200 mL of boiling water maintained at 100^ o^C in accordance with the specifications provided by the manufacturer. The resulting solution was allowed to cool to room temperature (37 ^o^C) prior to use. The composite discs were immersed in 15 mL of their respective solutions for 10 minutes/day for 30 days. 

The specimens were subjected to thermal cycling using a thermal cycler (Atico India, Ambala Cantt, Haryana, India). The thermal cycling process involved aging of the specimens, thereby simulating prolonged intraoral temperature fluctuations. The specimens were alternately immersed in thermal baths of hot (55 ^o^C) and cold (5 ^o^C) water with a predetermined dwell time of 30 seconds in each bath and a transfer interval of 10 seconds between baths. The temperature variation was meticulously maintained at ± 2 ^o^C throughout the procedure to ensure uniform thermal stress. A total of 5,000 thermocycles were performed during the thermocycling process, simulating approximately six months of clinical aging.

The discs were then mounted onto glass slides and transported to the laboratory for color analysis. Spectrophotometric evaluation was conducted using a spectrophotometer (Vita Easyshade 2.0, VITA Zahnfabrik, Germany). The device was calibrated against a grey background before each measurement using the manufacturer-provided calibration tile to ensure accuracy and reliability. The CIE L*a*b* values were documented at the baseline (prior to immersion) and subsequent to a duration of 30 days of immersion in the evaluated solutions. Quantification of color alteration (ΔE) was calculated using the following formula: ΔE = ½[(ΔL)² + (Δa)² + (Δb)²], where ΔE* represents the degree of color variation, l* denotes the luminance reflectance, a* signifies the red-green chromaticity coordinate, and b* indicates the yellow-blue chromaticity coordinate. Spectrophotometric assessments were conducted on three separate occasions at the centroid of each specimen, and the mean values of the L*, a*, and b* parameters were documented for subsequent analyses.

Statistical analysis

All statistical analyses were conducted using IBM SPSS Statistics for Windows, Version 23.0 (Released 2015; IBM Corp., Armonk, New York, United States). Descriptive statistics, including mean ± standard deviation and median with minimum-maximum ranges, were used to summarize continuous variables. The normality of the numerical data was assessed using the Kolmogorov-Smirnov and Shapiro-Wilk tests. An independent t-test was used to evaluate differences in color change (∆E) between Omnichroma and Vittra Unique. One-way analysis of variance (ANOVA) was performed for parametric comparisons among subgroups, followed by post hoc analysis using the Tukey test. The significance level was set at p < 0.05.

## Results

One-way ANOVA demonstrated a significant variation in staining resistance among the four subgroups (p = 0.001), rejecting the null hypothesis that there was no difference in mean color change between the groups. The highest mean color change was observed in the Vittra coffee subgroup (ΔE = 2.70 ± 0.56), suggesting that the Vittra composite exhibited the greatest susceptibility to staining when exposed to coffee. This was followed by Vittra chlorhexidine (ΔE = 2.05 ± 0.41), indicating that chlorhexidine also induced notable discoloration in Vittra, although to a lesser extent than coffee. In contrast, the Omnichroma subgroups displayed lower mean color changes, with Omnichroma coffee showing the least discoloration (ΔE = 1.25 ± 0.17) and Omnichroma chlorhexidine recording a slightly higher value (ΔE = 1.61 ± 0.37). These findings suggest that Omnichroma demonstrates superior staining resistance compared to Vittra, under both coffee and chlorhexidine exposure (Table 1).

**Table 1:** Comparison of mean color change between the subgroups using one way analysis of variance (ANOVA). *p value < 0.05: significant CHX: chlorhexidine, SEM: standard error of mean, Omnichroma (Tokuyama Dental, Tokyo, Japan), Vittra Unique (FGM Dental Group, Joinville, SC, Brazil)

Subgroups	Frequency (Percentage)	Mean ± SD	SEM	F stat	p value
Vitraa CHX	10 (25%)	2.05 ± 0.41	0.13	24.07	0.001*
Vitraa coffee	10 (25%)	2.70 ± 0.56	0.18		
Omnichroma CHX	10 (25%)	1.61 ± 0.37	0.12		
Omnichroma coffee	10 (25%)	1.25 ± 0.17	0.05		

The post hoc Tukey test confirmed that significant differences in staining resistance existed between subgroups. Vittra coffee demonstrated the highest susceptibility to staining, as evidenced by its significant mean differences compared with all other groups, particularly the largest gap with Omnichroma coffee (1.45, p = 0.001), highlighting the pronounced effect of coffee on Vittra. Conversely, Omnichroma coffee exhibited the least color change, with significantly lower ΔE values compared to both Vittra subgroups and Omnichroma chlorhexidine, suggesting superior resistance to coffee-induced staining. Vittra chlorhexidine showed intermediate staining, significantly higher than both Omnichroma groups, but lower than Vittra coffee (p = 0.008), indicating that chlorhexidine affects Vittra less severely than coffee. The significant difference between Omnichroma chlorhexidine and Omnichroma coffee (p = 0.011) further suggested that coffee had a milder staining impact on Omnichroma than chlorhexidine. These results revealed the overall superior performance of Omnichroma, particularly against coffee (Table 2).

**Table 2:** Intergroup pairwise for color staining using post hoc Tukey test. *p value < 0.05: significant CHX: chlorhexidine, MD: mean difference, CI: confidence interval, Omnichroma (Tokuyama Dental, Tokyo, Japan), Vittra Unique (FGM Dental Group, Joinville, SC, Brazil).

Pairwise comparison		MD	CI at 95%	t stat	p value
Vitraa CHX	Vitraa coffee	-0.65	-1.11 to -0.19	2.96	0.008*
Vitraa CHX	Omnichroma CHX	0.44	0.07 to 0.80	2.51	0.021*
Vitraa CHX	Omnichroma coffee	0.80	0.50 to 1.09	5.69	0.005*
Vitraa coffee	Omnichroma CHX	1.09	0.64 to 1.53	5.13	0.001*
Vitraa coffee	Omnichroma coffee	1.45	1.06 to 1.83	7.83	0.001*
Omnichroma CHX	Omnichroma coffee	0.36	0.084 to 0.63	2.79	0.011*

## Discussion

This in vitro study assessed the staining resistance of two commercially available single-shade universal composite resins, Omnichroma and Vittra Unique, exposed to two commonly encountered staining agents, coffee and 0.2% chlorhexidine. The results demonstrated a statistically significant difference in staining susceptibility between the materials, with Omnichroma exhibiting superior resistance compared to Vittra Unique across both staining solutions.

Omnichroma is a universal shade nanocomposite that features a simplified resin matrix composed primarily of urethane dimethacrylate (UDMA) and triethylene glycol dimethacrylate (TEGDMA). The filler system consists of uniform supranano spherical particles made of zirconia and silica, with an average size of approximately 260 nanometers. These fillers are uniquely designed to create structural color without the use of added pigments, allowing the composite to adapt to surrounding tooth shades based on light reflection and scattering. The total filler content is 79% by weight and 68% by volume. In contrast, Vittra Unique is a nanohybrid composite that contains a more conventional blend of monomers, including bisphenol A-glycidyl methacrylate (Bis-GMA), UDMA, and TEGDMA. This composition results in a resin matrix with relatively higher hydrophilicity, contributing to increased water sorption and potentially greater stain absorption over time. Vittra Unique achieves its chromatic blending effect through the incorporation of both organic and inorganic pigments and colorants, unlike Omnichroma’s pigment-free structural color approach [12].

This study employed coffee and chlorhexidine as staining agents, which are frequently utilized by both adult and younger populations on a daily basis and exhibit the propensity to discolor tooth-colored restorations [2,3]. The duration of immersion spanning 30 days was aligned with a prior comprehensive study that demonstrated that a 30-day immersion period represented the upper limit that could be feasibly implemented [14]. This finding corroborated the evaluation conducted by Ertas et al., who posited that a 30-day duration is tantamount to two years and six months of clinical aging (with 24 hours of in vitro staining corresponding to approximately one month of in vivo exposure) [15].

The findings of this study indicated that Vittra Unique exhibited greater staining susceptibility than Omnichroma, with the highest color change (ΔE = 2.70 ± 0.56) observed in the Vittra coffee subgroup. This suggests that Vittra's formulation may be more prone to absorbing stain-inducing compounds present in coffee. In contrast, Omnichroma demonstrated the least color change (ΔE = 1.25 ± 0.17) when exposed to coffee, suggesting a superior ability to resist extrinsic staining. Chlorhexidine, while also inducing discoloration, had a less pronounced effect than coffee, with Omnichroma. This finding was in agreement with a study by Tepe et al., who found that Omnichroma exhibited the least color change at all observed time points [16].

Staining susceptibility in composite resins is influenced by various factors, including the resin matrix composition, filler type and size, polymerization efficiency, and surface roughness [17]. The lower staining susceptibility of Omnichroma may be attributed to its unique Smart Chromatic Technology, which utilizes structural color rather than pigment-based shade matching. This technology relies on uniform spherical fillers that scatter and reflect the surrounding light to blend seamlessly with the tooth structures. As a result, they may exhibit lower pigment absorption and reduced susceptibility to extrinsic staining [10].

Conversely, Vittra Unique, which incorporates a combination of organic and inorganic pigments for shade adaptation, may be more prone to stain absorption owing to the presence of colorants in its formulation [18]. Common organic pigments include azo dyes, phthalocyanines, and quinacridones, while inorganic pigments such as titanium dioxide (TiO₂) and iron oxides contribute to its optical properties. These pigments enhance esthetic blending but may increase susceptibility to extrinsic staining, particularly in the coffee subgroup, which showed higher ΔE values. Furthermore, Vittra Unique’s resin matrix contains a relatively high proportion of hydrophilic monomers, such as TEGDMA. This contributes to greater hydrophilicity, which increases water sorption and, consequently, stain absorption over time. Materials with higher hydrophilicity tend to exhibit less color stability, especially when exposed to chromogenic substances in beverages like coffee and red wine.

Coffee, a widely consumed beverage, is known for its high staining potential owing to the presence of tannins and chromogens, which can readily adhere to resin surfaces and penetrate microvoids within the composite [19]. As articulated by Ozkanoglu and Akin, composite materials submerged in coffee demonstrated a greater degree of chromatic alteration than those immersed in tea or cola throughout a duration of six weeks dedicated to staining [20]. The higher staining observed in the Vittra coffee subgroup aligns with studies that have highlighted coffee as a major extrinsic staining agent for resin-based materials [19,20].

Chlorhexidine, commonly used in oral rinses, also caused significant discoloration, particularly in the Vittra Unique (ΔE = 2.05 ± 0.41). This is consistent with previous studies indicating that prolonged exposure to chlorhexidine can result in yellow-brown discoloration owing to the precipitation of pigmented by-products on composite surfaces [21]. The fact that Omnichroma demonstrated lower ΔE values in both staining solutions suggests its superior resistance to stain uptake.

Surface roughness plays a crucial role in staining resistance because a smoother surface reduces the likelihood of stain adsorption [22]. In the current study, all specimens underwent standardized polishing using Sof-Lex disks to ensure uniform surface smoothness. Despite this, differences in staining were still observed between the materials, suggesting that intrinsic factors, such as polymerization efficiency and resin composition, played a more significant role in stain resistance. The polymerization process can also influence the extent of stain uptake. Inadequate polymerization can result in residual monomers that contribute to increased water sorption and color instability [23]. The use of a high-intensity LED curing unit with a standardized curing protocol in the current study ensured optimal polymerization and minimized discrepancies related to curing efficiency.

Clinical implications

The results of this study have direct clinical relevance as esthetic longevity is a primary concern for both clinicians and patients. The superior resistance of Omnichroma to staining suggests that it may be the preferred choice for restorations in highly visible areas, particularly in patients with high coffee consumption or those using chlorhexidine rinses. However, despite its higher staining susceptibility, Vittra Unique may still be suitable for esthetic restorations, provided that patients adhere to proper oral hygiene practices and minimize their exposure to staining agents. Additionally, routine polishing and maintenance of composite restorations are essential to mitigate staining over time [24]. Clinicians should educate patients on the potential discoloration effects of dietary and oral hygiene habits, emphasizing the importance of regular professional cleaning to maintain the esthetics of composite restorations.

Limitations and future recommendations

While this study provides valuable insights into the staining resistance of Omnichroma and Vittra Unique, certain limitations must be acknowledged. First, this was an in vitro study, and the results may not fully replicate intraoral conditions where factors such as salivary flow, enzymatic activity, and mechanical forces can influence staining dynamics. Future in vivo studies with a long-term follow-up are necessary to validate these findings. Second, the staining period was limited to 30 days, which may not accurately represent the long-term color stability of these materials. Extended study durations with periodic assessments could provide a more comprehensive understanding of the progressive staining behavior of these composites. Finally, further research exploring additional staining agents, such as red wine, tea, and nicotine, could offer a broader perspective on the color stability of single-shade universal composites. Investigating the impact of different polishing protocols and surface sealants on strain resistance could also provide valuable clinical guidance for optimizing restorative longevity.

## Conclusions

Omnichroma exhibited superior staining resistance compared to Vittra Unique when exposed to both coffee and chlorhexidine over a 30-day period. Of the two staining agents, coffee caused the highest degree of discoloration, with Vittra Unique being the most affected. The highest color change was observed in the Vittra coffee subgroup, indicating that this composite material was more susceptible to staining than Omnichroma. Chlorhexidine also contributed to the discoloration, although its effect was less pronounced than that of coffee. In contrast, Omnichroma demonstrated significantly lower color changes, particularly in the coffee subgroup, suggesting its enhanced ability to resist external staining. These findings hold clinical significance in the selection of restorative materials, as Omnichroma may be a more suitable option for patients with high exposure to staining agents such as coffee or chlorhexidine-based mouthwashes.

## References

[1] Tin-Oo MM, Saddki N, Hassan N (2011). Factors influencing patient satisfaction with dental appearance and treatments they desire to improve aesthetics. BMC Oral Health.

[2] Hattab FN, Qudeimat MA, al-Rimawi HS (1999). Dental discoloration: an overview. J Esthet Dent.

[3] Eriksen HM, Nordbø H (1978). Extrinsic discoloration of teeth. J Clin Periodontol.

[4] Hajdu AI, Dumitrescu R, Balean O (2024). Enhancing esthetics in direct dental resin composite: investigating surface roughness and color stability. J Funct Biomater.

[5] Pietrokovski Y, Zeituni D, Schwartz A, Beyth N (2022). Comparison of different finishing and polishing systems on surface roughness and bacterial adhesion of resin composite. Materials (Basel).

[6] Rohym S, Tawfeek HE, Kamh R (2023). Effect of coffee on color stability and surface roughness of newly introduced single shade resin composite materials. BMC Oral Health.

[7] Poppolo Deus F, Ouanounou A (2022). Chlorhexidine in dentistry: pharmacology, uses, and adverse effects. Int Dent J.

[8] Fathima JN, Hashir MM, Padmanabhan K (2024). Spectrophotometric evaluation of color stability of composite resin after exposure to cold drinks: an in vitro study. J Conserv Dent Endod.

[9] Seghi RR, Johnston WM, O'Brien WJ (1989). Performance assessment of colorimetric devices on dental porcelains. J Dent Res.

[10] Baghizadeh S, Tabari K, Abbasi K, Tabatabaei SF, Heshmat H (2024). Assessing shade matching capability of Omnichroma, a single shade composite in posterior restorations: an in vitro study. J Med Life.

[11] Ruiz-López J, Mariano da Rocha BG, Zemolin NA, Altenhofen CS, Durand LB, Pérez MM (2024). Visual evaluation of the color adjustment of single-shade and group shade resin composites in restorations with different cavity configurations. J Dent.

[12] Forabosco E, Josic U, Consolo U, Generali L, D'Alessandro C, Breschi L, Checchi V (2025). Color match of single-shade versus multi-shade resin composites: a systematic review with meta-analysis. J Esthet Restor Dent.

[13] Özdemir B, Kurucu Karadeniz BK, Özdemir SB, Akbulut Ö (2024). How the translucency and color stability of single-shade universal resin composites are affected by coffee?. Curr Res Dent Sci.

[14] Nasim I, Neelakantan P, Sujeer R, Subbarao CV (2010). Color stability of microfilled, microhybrid and nanocomposite resins--an in vitro study. J Dent.

[15] Ertaş E, Güler AU, Yücel AC, Köprülü H, Güler E (2006). Color stability of resin composites after immersion in different drinks. Dent Mater J.

[16] Tepe H, Celiksoz O, Yaman BC (2025). Evaluation of color stability in single-shade composite resins using spectrophotometer and cross-polarized mobile photography. BMC Oral Health.

[17] Cinelli F, Scaminaci Russo D, Nieri M, Giachetti L (2022). Stain susceptibility of composite resins: pigment penetration analysis. Materials (Basel).

[18] Santana ML, Livi GJ, Faria-E-Silva AL (2024). Color discrepancy of single-shade composites at different distances from the interface measured using cell phone images. Restor Dent Endod.

[19] Karaman E, Tuncer D, Firat E, Ozdemir OS, Karahan S (2014). Influence of different staining beverages on color stability, surface roughness and microhardness of silorane and methacrylate-based composite resins. J Contemp Dent Pract.

[20] Ozkanoglu S, G Akin EG (2020). Evaluation of the effect of various beverages on the color stability and microhardness of restorative materials. Niger J Clin Pract.

[21] Ernst CP, Prockl K, Willershausen B (1998). The effectiveness and side effects of 0.1% and 0.2% chlorhexidine mouthrinses: a clinical study. Quintessence Int.

[22] Lu H, Roeder LB, Lei L, Powers JM (2005). Effect of surface roughness on stain resistance of dental resin composites. J Esthet Restor Dent.

[23] Unsal KA, Karaman E (2022). Effect of additional light curing on colour stability of composite resins. Int Dent J.

[24] Vishwanath S, Kadandale S, Kumarappan SK, Ramachandran A, Unnikrishnan M, Nagesh HM (2022). Finishing and polishing of composite restoration: assessment of knowledge, attitude and practice among various dental professionals in India. Cureus.

